# Supramolecular one-dimensional conducting nanofibers from a *C*_3_-symmetric tetrathiafulvalene derivative

**DOI:** 10.3762/bjnano.17.63

**Published:** 2026-07-10

**Authors:** Yoko Tatewaki, Fumiya Hirose, Sadafumi Nishihara, Tomoyuki Akutagawa, Takayoshi Nakamura, Tsuyoshi Minami

**Affiliations:** 1 Department of Applied Chemistry, Tokyo University of Agriculture and Technology, 2-24-16 Nakacho, Koganei-shi, Tokyo, 184-8588 Japan; 2 Graduate School of Advanced Science and Engineering, Hiroshima University, 1-3-1, Kagamiyama, Higashi-hiroshima 739-8526, Japan; 3 Institute of Multidisciplinary Research for Advanced Materials (IMRAM), Tohoku University, Sendai 980-8577, Japan; 4 Research Institute for Electronic Science, Hokkaido University, Sapporo, Japan; 5 Institute of Industrial Science, The University of Tokyo, 4-6-1 Komaba, Meguro-ku, Tokyo 153-8505, Japan

**Keywords:** charge-transfer complex, conducting nanofibers, one-dimensional nanostructures, supramolecular self-assembly, tetrathiafulvalene

## Abstract

This study reports the design and synthesis of a *C*_3_-symmetric discotic tetrathiafulvalene (TTF) derivative, MeS-TTF-Ts, as a molecular platform for the self-assembly of one-dimensional conductive nanostructures. The molecular architecture contains three TTF units radially arranged around a rigid *C*_3_-symmetric core, enabling the formation of highly ordered one-dimensional assemblies through cooperative π–π stacking and directional intermolecular interactions. Solution casting of MeS-TTF-Ts produced nanorod and nanofiber structures with morphologies strongly dependent on the solvent conditions. AFM and SEM observations revealed that distinct supramolecular structures were formed depending on the solvent and substrate used, suggesting that molecular interactions and assembly processes play important roles in determining the resulting morphologies. Furthermore, MeS-TTF-Ts was combined with the strong electron acceptor F4TCNQ to form a charge-transfer complex, (MeS-TTF-Ts)(F4TCNQ)_3_, which yielded well-defined one-dimensional nanofibers. UV–vis and FTIR spectroscopic analyses confirmed substantial charge transfer from MeS-TTF-Ts to F4TCNQ through the formation of F4TCNQ^−^ and TTF^+^ species. These results indicate the formation of a highly charge-transferred donor–acceptor assembly in the nanofibers. Electrical conductivity measurements revealed that the (MeS-TTF-Ts)(F4TCNQ)_3_ nanofibers exhibit a conductivity of 1.24 × 10^−2^ S·cm^−1^. These findings demonstrate that rational molecular design combined with controlled supramolecular self-assembly provides an effective strategy for constructing one-dimensional conductive nanostructures based on organic charge-transfer systems.

## Introduction

Supramolecular gels have been developed as organized soft materials that form anisotropic nanostructures such as ribbons and fibers within their networks [1–2]. These materials have attracted considerable attention in the fields of materials science and nanotechnology because of their diverse functionalities, including luminescence, sensing, charge transport, and drug delivery [3–6]. Supramolecular gels are formed through bottom-up self-assembly driven by weak intermolecular interactions such as hydrogen bonding, π–π stacking, and donor–acceptor interactions, resulting in three-dimensional interconnected nanofibrous networks [7–8]. Such one-dimensional nanostructures can be precisely controlled in terms of morphology and physical properties through molecular design, making them promising platforms for the development of functional nanomaterials [9–10].

In particular, when electroactive molecules are employed, one-dimensional structures formed through self-assembly are expected to be applicable as nanowire materials in molecular electronic devices. Among them, tetrathiafulvalene (TTF) derivatives are representative redox-active molecules capable of forming charge-transfer complexes, and their charge-transfer salts have long been known to exhibit high electrical conductivity [11–14].

In recent years, TTF derivatives functionalized with amide groups or long alkyl chains have been reported to form highly ordered one-dimensional stacked structures through cooperative hydrogen bonding and π–π stacking interactions, leading to the formation of nanowires via self-assembly [15–20]. These assemblies can be partially oxidized through charge-transfer interactions with iodine or strong electron acceptors, thereby generating TTF radical cation species that serve as charge transport pathways. As a result, their electrical properties can be tuned from semiconducting to metallic behavior, depending on the doping level [21–24].

The realization of nanoscale conductive structures requires precise control of intermolecular interactions and rational molecular design capable of directing the spontaneous formation of one-dimensional stacked assemblies [25–28]. In particular, for molecules incorporating redox-active units, it has been reported that one-dimensional structures formed through self-assembly can directly function as charge transport pathways [29–30].

*C*_3_-symmetric discotic molecules, which possess a rigid central core and three-directional interaction sites, are well known as molecular platforms that readily form highly ordered columnar stacking structures [31–33]. Furthermore, expansion of the π-conjugated framework enhances electronic interactions between stacked units, and the cooperative incorporation of noncovalent interactions such as directional hydrogen bonding enables the construction of stable one-dimensional nanostructures while facilitating efficient linear stacking of conductive units [34–36].

## Experimental

### General

Nuclear magnetic resonance (NMR) spectra were recorded on a JEOL ECX-400 spectrometer operating at 400 MHz for ^1^H NMR and 100.6 MHz for ^13^C NMR. Chemical shifts are reported in parts per million (ppm) relative to external tetramethylsilane (TMS) and are given downfield.

Mass spectrometry measurements were carried out using a JEOL JMS instrument. Elemental analyses were performed at the Research Support Center, Okayama University.

### Materials

Mercury(II) acetate, triethyl phosphite, lithium bromide (anhydrous), lithium hydroxide monohydrate, oxalyl chloride, and pyridine were purchased from Kanto Chemical Co., Inc. 2,6-Diaminopyridine and 1,3,5-benzenetricarbonyl trichloride were purchased from Tokyo Chemical Industry Co., Ltd. (TCI). All reagents were used as received without further purification unless otherwise noted.

### Synthesis of compounds

#### 4,5-Bis(methylthio)-1,3-dithiole-2-thione (**2**)

Iodomethane (790 mg, 5.56 mmol) was added to a solution of zinc chelate **1** (1.000 g, 1.39 mmol) in acetone (25 mL). The reaction mixture was stirred at 25 °C overnight. After removal of the solvent under reduced pressure, the residue was extracted with chloroform and washed with brine. The organic layer was dried over MgSO_4_, filtered, and concentrated under reduced pressure. The crude product was purified by recrystallization from acetone to afford compound **2** as yellow needle crystals. Yield 401.0 mg (63.7%) [37–38]. ^1^H NMR (400 MHz, CDCl_3_) δ 2.50 (s, 6H); ^13^C NMR (100.6 MHz, CDCl_3_) δ 211.0, 136.0, 19.4; MS (FAB) *m*/*z*: [M + H]^+^ calcd for C_5_H_6_S_5_, 226.91; found, 227.

#### Dimethyl 1,3-dithiole-2-oxo-4,5-dicarboxylate (**3**)

Mercury(II) acetate (3.824 g, 12.0 mmol) was added to a solution of dimethyl 1,3-dithiole-2-thioxo-4,5-dicarboxylate (999.6 mg, 3.99 mmol) in chloroform (40 mL). The reaction mixture was stirred at 25 °C for 1 h. The mixture was filtered through Celite and washed with chloroform. The solvent was removed under reduced pressure, and the residue was purified by column chromatography (chloroform) to afford compound **3** as a white solid. Yield 898.7 mg (96.1%) [39–40]. ^1^H NMR (400 MHz, CDCl_3_) δ 3.91 (s, 6H); ^13^C NMR (100.6 MHz, CDCl_3_) δ 186.4, 159.2, 129.1, 53.6; MS (FAB) *m*/*z*: [M + H]^+^ calcd for C_7_H_6_O_5_S_2_, 234.97; found, 235.

#### MeS-TTF(COOMe)_2_ (**4**)

Compounds **2** (358.6 mg, 1.58 mmol) and **3** (373.4 mg, 1.59 mmol) were dissolved in a mixture of triethyl phosphite (10 mL) and toluene (10 mL). The reaction mixture was refluxed at 110 °C for 2 h. After removal of the solvent under reduced pressure, toluene (100 mL) was added to the residue and evaporated under reduced pressure. This procedure was repeated several times. The crude product was purified by column chromatography (chloroform) to afford compound **4** as a dark brown solid. Yield 381.0 mg (57.9%) [38,41–42]. ^1^H NMR (400 MHz, CDCl_3_) δ 3.85 (s, 6H), 2.43 (s, 6H); ^13^C NMR (100.6 MHz, CDCl_3_) δ 159.9, 132.0, 127.7, 112.5, 99.9, 53.4, 19.3; MS (FAB) *m*/*z*: [M + H]^+^ calcd for C_12_H_12_O_4_S_6_, 411.91; found; 412.

#### MeS-TTF-COOMe (**5**)

A solution of **4** (360.0 mg, 0.873 mmol) and lithium bromide (1.600 g, 18.4 mmol) in dry DMF (15 mL) was stirred at 85 °C for 3.5 h. After cooling to room temperature, brine was added, and the mixture was extracted with ethyl acetate. The combined organic layers were washed with brine, dried over MgSO_4_, filtered, and concentrated under reduced pressure. The crude product was purified by column chromatography (chloroform) to afford compound **5** as a brown solid. Yield 130.8 mg (42.3%) [43–44]. ^1^H NMR (400 MHz, CDCl_3_) δ 7.34 (s, 1H), 3.85 (s, 3H), 2.43 (s, 6H); ^13^C NMR (100.6 MHz, CDCl_3_) δ 159.7, 132.0, 127.9, 127.1, 112.9, 109.4, 52.7, 19.2; MS (FAB) *m*/*z*: [M + H]^+^ calcd for C_10_H_10_O_2_S_6_, 353.90; found, 354.

#### MeS-TTF-COOH (**6**)

An aqueous solution of lithium hydroxide (82.0 mg, 1.95 mmol) was added to a solution of **5** (130.8 mg, 0.369 mmol) in 1,4-dioxane (8.5 mL). The reaction mixture was stirred at room temperature overnight. The mixture was then acidified with 5 M HCl (ca. 1.0 mL), during which the solution turned red. After stirring for 30 min, diethyl ether (6.0 mL) and water (2.0 mL) were added to form a biphasic system. Additional 5 M HCl was added until the pH reached 1–2. The layers were separated, and the aqueous phase was extracted with diethyl ether. The combined organic layers were dried over MgSO_4_, filtered, and concentrated under reduced pressure to afford compound **6** as a red solid. Yield 115.7 mg (92.1%) [44–45]. ^1^H NMR (400 MHz, DMSO-*d*_6_) δ 11.8–14.6 (br, 1H), 7.71 (s, 1H), 2.43 (s, 6H); ^13^C NMR (100.6 MHz, DMSO-*d*_6_) δ 160.3, 133.0, 128.8, 126.4, 113.8, 106.8, 18.6; MS (FAB) *m*/*z*: [M + H]^+^ calcd for C_9_H_8_O_2_S_6_, 339.88; found, 340.

#### MeS-TTF-COCl (**7**)

Oxalyl chloride (113.7 μL, 1.32 mmol) was added dropwise to a solution of **6** (113.0 mg, 0.332 mmol) and pyridine (0.45 μL) in dry THF (10 mL). The reaction mixture was stirred at 45 °C for 2 h. The solvent and excess oxalyl chloride were removed under reduced pressure, and the residue was washed with pentane to afford compound **7** as a purple solid in quantitative yield. The product was used in the next step without further purification [46–47].

#### MeS-TTF-CONHPy (**8**)

A solution of **7** (47.8 mg, 0.133 mmol) in dry THF was added to a solution of 2,6-diaminopyridine (16.80 mg, 0.154 mmol) and triethylamine (24.0 μL, 0.172 mmol) in dry THF (1.0 mL) at 0 °C. The reaction mixture was stirred at 0 °C for 2 h and then at room temperature overnight. The solvent was removed under reduced pressure, and the residue was purified by column chromatography (chloroform/methanol = 47:3) to afford compound **8** as a red-brown solid. Yield 25.0 mg (43.5%) [46]. ^1^H NMR (400 MHz, CDCl_3_) δ 7.89 (s, 1H), 7.50 (m, *J* = 24.8 Hz, 2H), 7.24 (s, 1H), 6.30 (d, *J* = 7.6 Hz, 1H), 4.40 (s, 2H), 2.43 (s, 6H); ^13^C NMR (100.6 MHz, CDCl_3_) δ 157.0, 148.9, 140.4, 132.2, 128.0, 127.2, 127.1, 111.9, 110.4, 105.0, 103.6, 19.2; MS (FAB) *m*/*z*: [M + H]^+^ calcd for C_14_H_13_N_3_OS_6_, 430.94; found, 431.

#### MeS-TTF-Ts

A solution of **8** (25.0 mg, 57.9 μmol) and triethylamine (7.65 μL, 54.9 μmol) in dry THF (1.0 mL) was cooled to 0 °C. A solution of trimesoyl chloride (5.33 mg, 20.1 μmol) in dry THF (1.0 mL) was added dropwise to the stirred solution at 0 °C. The reaction mixture was stirred at 0 °C for 2 h and then at room temperature overnight. Hexane was added to the mixture to induce precipitation, and the resulting solid was collected and washed with ethyl acetate, methanol, and hexane to afford MeS-TTF-Ts as a brown solid. Yield 14.65 mg (55.9%) [48–49]. ^1^H NMR (400 MHz, DMSO-*d*_6_) δ 10.74 (m, *J* = 24.0 Hz, 3H), 8.67 (t, *J* = 13.6 Hz, 3H), 8.05 (d, *J* = 7.6 Hz, 3H), 7.93 (m, *J* = 15.6 Hz, 6H), 7.67 (m, *J* = 4.8 Hz, 3H), 2.45 (s, 18H); ^13^C NMR (100.6 MHz, DMSO-*d*_6_) δ 157.8, 150.2, 149.7, 149.4, 140.4, 134.6, 133.2, 131.7, 128.1, 126.1, 112.8, 106.0, 90.7, 79.2, 18.6; FTIR (ATR, νmax/cm^−1^): 2970, 2930, 2873, 1698, 1584, 1512, 1445, 1292, 1236, 773, 699. The FTIR spectrum of MeS-TTF-Ts is shown in Figure S1 in Supporting Information File 1. MS (APCI) *m*/*z*: [M + H]^+^ calcd for C_51_H_39_N_9_O_6_S_18_, 1449.80; found, 1449.8082; Anal. calcd for C_51_H_39_N_9_O_6_S_18_: C, 42.21; H, 2.71; N, 8.69; found: C, 42.12; H, 2.66; N, 8.63.

### Measurements

#### AFM measurements

The structures formed on mica substrates were observed using an atomic force microscopy (AFM) system (Bruker Multi Mode 8). Measurements were performed in non-contact mode using a silicon cantilever with a spring constant of approximately 0.8 N·m^−1^.

#### Optical spectra

UV–vis absorption spectra were recorded in the range of 300–1200 nm using a JASCO V-650 spectrophotometer. A mixed solvent of dimethyl sulfoxide (DMSO) and acetonitrile was used for the measurements. Fourier-transform infrared (FTIR) spectra were recorded using a JASCO FT/IR-4200 spectrometer. Spectra were obtained either by the ATR method or from cast films deposited on quartz substrates, depending on the sample form.

#### Conductivity measurements

Electrical conductivity was measured by a direct current (DC) two-probe method. Gold electrodes with a thickness of 100 nm were deposited on mica substrates by vacuum evaporation. Electrical contacts were made by attaching gold wires (25 μm in diameter) using silver paste.

Temperature-dependent DC conductivity was measured using a Keithley 6517 electrometer, with an applied voltage in the range of −100 to +100 V. The electrode gap and electrode length were 60 μm and 0.6 cm, respectively.

## Results and Discussion

### Synthesis

**Scheme 1:**
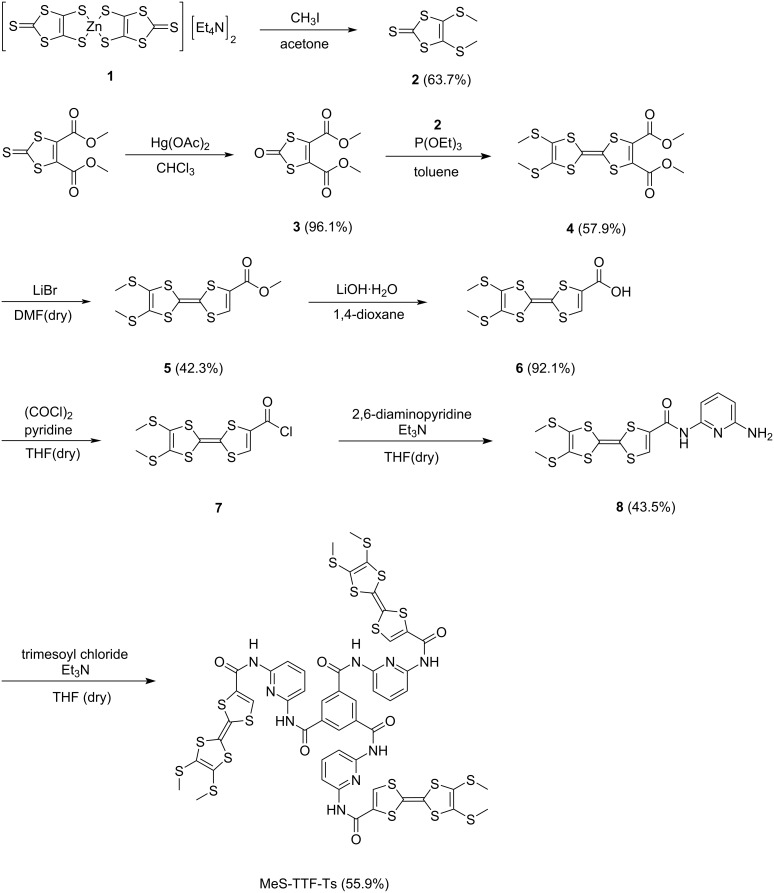
Synthesis of *C*_3_-symmetric MeS-TTF-Ts.

The synthetic strategy employed for the preparation of MeS-TTF-Ts in this study is similar to that reported by John D. Wallis and co-workers [50–51]. In the final step, an acylation reaction between compound **7** and compound **8** was carried out (Scheme 1).

### Formation of nanofibers from *C*_3_*-*symmetric MeS-TTF-Ts and the (MeS-TTF-Ts)(F4TCNQ)_3_ charge-transfer complex

In order to form conductive fibers using MeS-TTF-Ts, it was necessary to identify suitable conditions for controlling the formation of supramolecular structures. Atomic force microscopy (AFM) observations of cast films of MeS-TTF-Ts revealed the formation of well-defined one-dimensional nanostructures with relatively uniform morphology.

**Figure 1:**
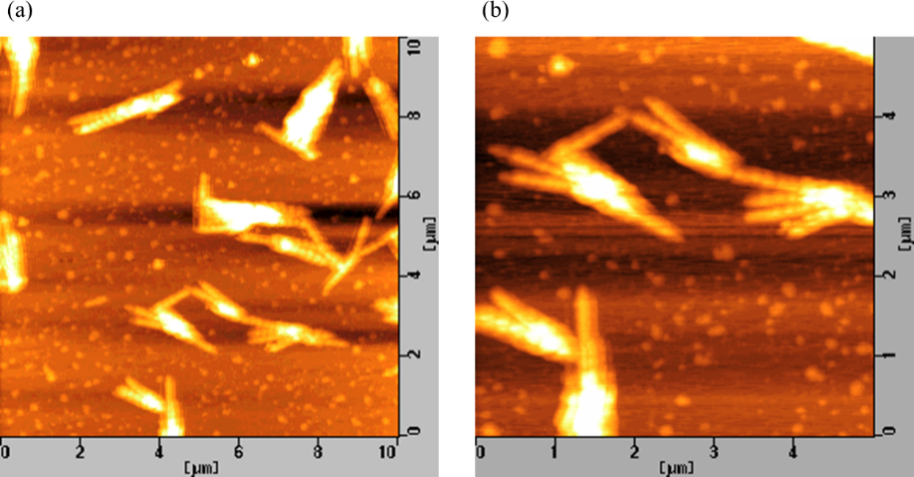
AFM images of nanorods composed of *C*_3_-symmetric MeS-TTF-Ts deposited from DMF on a mica substrate. (a) 10 μm × 10 μm; (b) 5 μm × 5 μm.

Specifically, dimethylformamide (DMF) solution of MeS-TTF-Ts (6 mM) was dripped onto a mica substrate, and the solvent was allowed to evaporate, resulting in the formation of nanorod-like structures with a width of 100 nm, a height of 30 nm, and a length of 3 μm (Figure 1). These nanorods were observed to be distributed across the substrate without significant aggregation, suggesting that the self-assembly process proceeds in a controlled manner under these conditions. The formation of such anisotropic nanostructures is attributed to directional π–π stacking interactions between the TTF cores, together with the cooperative effect of intermolecular interactions, which promote one-dimensional growth along a preferred direction. These results indicate that the solvent casting process in DMF provides suitable conditions for inducing ordered supramolecular organization of MeS-TTF-Ts.

**Figure 2:**
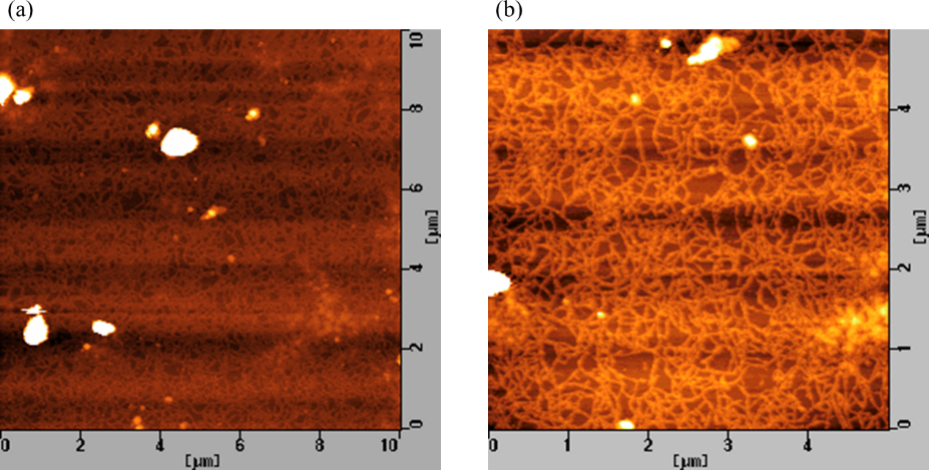
AFM images of nanofibers composed of *C*_3_-symmetric MeS-TTF-Ts deposited from DMSO on a mica substrate. (a) 10 μm × 10 μm; (b) 5 μm × 5 μm.

When the casting solvent was changed to DMSO at the same concentration, a mesh-like structure composed of interconnected fibers with different sizes was observed (Figure 2). The fibers exhibited dimensions of approximately 50 nm in width, 5 nm in height, and lengths exceeding 10 μm. Compared with the nanorod structures obtained from DMF, a markedly different morphology was observed in the DMSO system, highlighting the important role of solvent conditions in determining the self-assembled structures. Although the detailed mechanism of the assembly formation remains unclear, the observed differences in morphology may arise from differences in intermolecular interactions and molecular packing during the self-assembly process. In particular, the formation of different polymorphic assembly states may contribute to the distinct morphologies observed in DMF and DMSO systems. These results suggest that solvent-dependent molecular organization strongly influences the resulting supramolecular structures. Internal structures were also observed within these assemblies, suggesting that the fibers are composed of bundles of thinner fibrils forming a hierarchical structure. Such hierarchical organization is considered to arise from directional π–π stacking interactions between the TTF cores together with cooperative intermolecular interactions, which promote lateral association of one-dimensionally grown fibrils. However, due to the highly entangled nature of the fibrous network, precise statistical evaluation was difficult.

**Figure 3:**
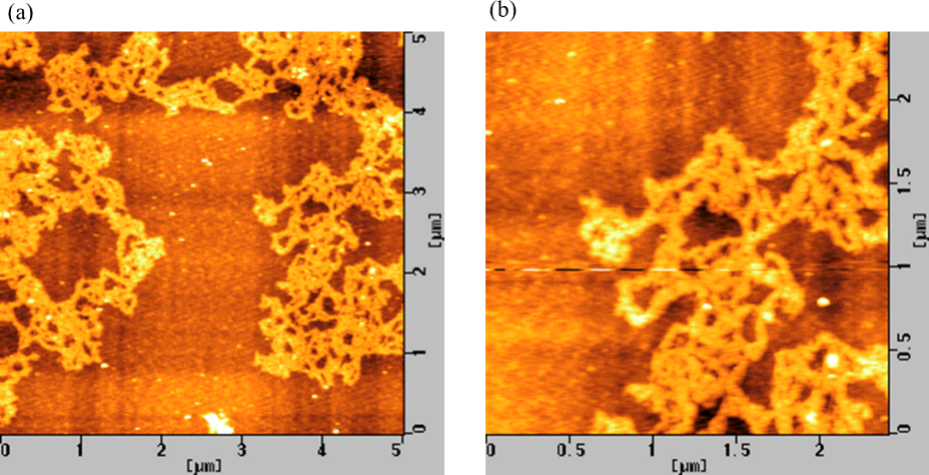
AFM images of nanofibers composed of the *C*_3_***-***symmetric (MeS-TTF-Ts)(F4TCNQ)_3_ charge-transfer complex deposited from a mixed solvent of DMSO and acetonitrile on a mica substrate. (a) 5 μm × 5 μm; (b) 2.5 μm × 2.5 μm.

A charge-transfer complex solution of (MeS-TTF-Ts)(F4TCNQ)_3_ was prepared by mixing a DMSO solution of the donor molecule MeS-TTF-Ts with an acetonitrile solution of the acceptor molecule F4TCNQ at a molar ratio of 1:3. AFM observations of the cast films of the resulting (MeS-TTF-Ts)(F4TCNQ)_3_ solution revealed the formation of well-defined one-dimensional nanofiber structures. Specifically, a mixed solution of MeS-TTF-Ts in DMSO and F4TCNQ in acetonitrile was prepared at a final concentration of 6 mM and dripped onto a mica substrate, followed by solvent evaporation. As a result, nanofibers with a width of 140 nm, a height of 6 nm, and lengths exceeding 5 μm were formed (Figure 3). These fibers were continuously distributed over the substrate, forming a relatively uniform network structure.

Furthermore, compared with the nanofibers formed from MeS-TTF-Ts alone, a significant increase in fiber diameter was observed. This result suggests that the introduction of F4TCNQ induces donor–acceptor charge-transfer interactions, leading to the formation of one-dimensional stacked structures composed of MeS-TTF-Ts and F4TCNQ. These results suggest that charge-transfer interactions enhance intermolecular interactions, thereby promoting the formation of thicker fibrous structures.

In contrast, when a mixed solution of MeS-TTF-Ts in DMF and F4TCNQ in acetonitrile was prepared at a final concentration of 6 mM and cast onto a mica substrate followed by solvent evaporation, no nanofiber structures were formed, and aggregated structures were observed instead. These aggregates exhibited a relatively uniform height of 140 nm. This result suggests that under these mixed solvent conditions, one-dimensional self-assembly does not proceed efficiently and the molecules tend to aggregate in a disordered manner. Furthermore, as observed for MeS-TTF-Ts alone, the morphology of the molecular assemblies in the (MeS-TTF-Ts)(F4TCNQ)_3_ system is strongly dependent on the solvent conditions. The observed differences may originate from variations in molecular organization, intermolecular interactions, and molecular packing during the self-assembly process. In other words, variations in solvent conditions lead to a competition between one-dimensional anisotropic growth and disordered aggregation, resulting in marked differences in the observed morphologies.

In addition to AFM observations, scanning electron microscopy (SEM) measurements were performed for representative samples deposited on HOPG substrates. Representative SEM images are provided in Supporting Information File 1 (Figures S2–S4). The morphologies observed by SEM differed from those obtained by AFM on mica substrates, showing more aggregated structures. This difference may originate from the distinct surface properties of the substrates used for the measurements. Although the detailed mechanism remains unclear, these results suggest that the self-assembly behavior of MeS-TTF-Ts and its charge-transfer complex is influenced by the substrate surface as well as the solvent conditions.

### Optical spectra of nanofibers formed from MeS-TTF-Ts and the (MeS-TTF-Ts)(F4TCNQ)_3_ charge-transfer complex

**Figure 4:**
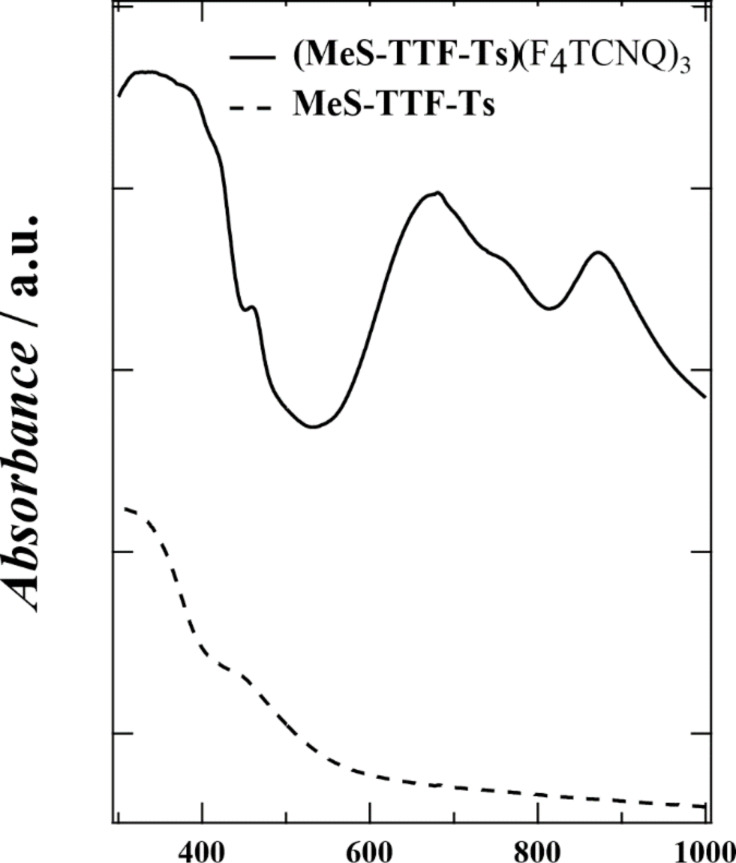
UV–vis spectra of nanofiber films on quartz substrates formed from MeS-TTF-Ts and the (MeS-TTF-Ts)(F4TCNQ)_3_ charge-transfer complex.

Figure 4 shows the UV–vis spectra of nanofiber cast films deposited on quartz substrates. The samples were prepared by drop-casting a DMSO solution of MeS-TTF-Ts and a mixed solution of MeS-TTF-Ts in DMSO and F4TCNQ in acetonitrile onto quartz substrates, followed by solvent evaporation. The final concentration of each solution was adjusted to 6 mM.

For the (MeS-TTF-Ts)(F4TCNQ)_3_ nanofibers, characteristic absorption bands were observed at 682 and 875 nm. These absorption bands are in good agreement with those previously reported for intramolecular transitions of the F4TCNQ radical anion (F4TCNQ^−^). In addition, the absorption bands observed at 462 and 333 nm are assigned to intramolecular transitions of the TTF radical cation (TTF^+^) and F4TCNQ^−^, respectively [13–14,52–55]. The appearance of these absorption bands suggests the formation of a charge-transfer state through electron transfer from the donor MeS-TTF-Ts to the acceptor F4TCNQ.

**Figure 5:**
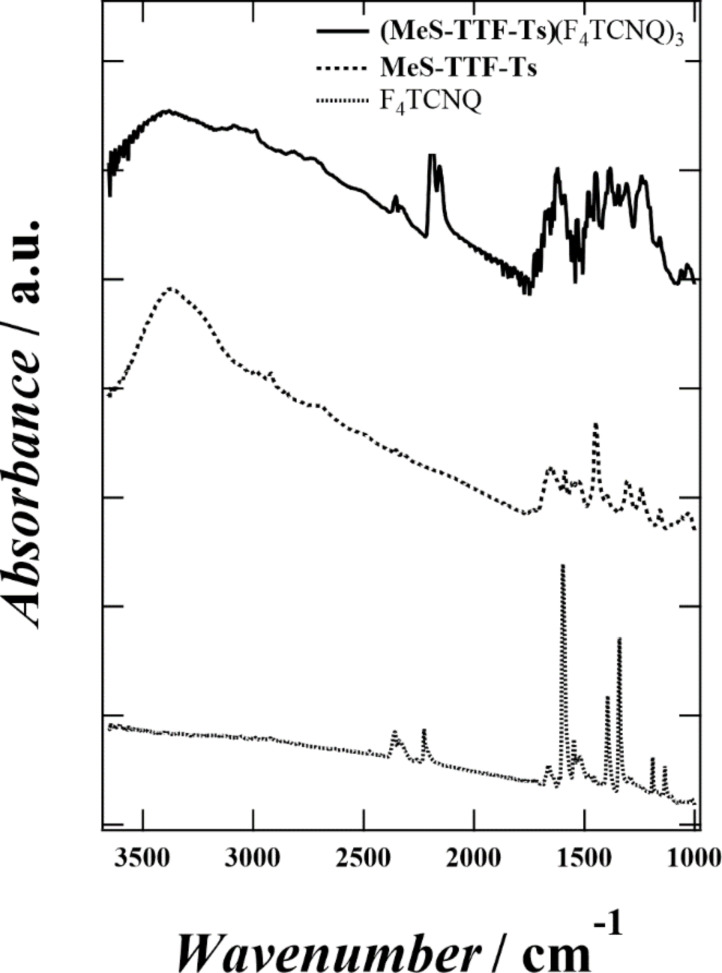
FTIR spectra of neutral F4TCNQ and the (MeS-TTF-Ts)(F4TCNQ)_3_ charge-transfer complex thin film deposited on a CaF_2_ substrate.

Figure 5 shows the FTIR spectra of neutral F4TCNQ and the (MeS-TTF-Ts)(F4TCNQ)_3_ charge-transfer complex thin film deposited on a CaF_2_ substrate. The thin film was prepared by drop-casting a mixed solution of MeS-TTF-Ts in DMSO and F4TCNQ in acetonitrile onto a CaF_2_ substrate, followed by solvent evaporation. The final concentration of the solution was adjusted to 6 mM.

To further investigate the electronic state of the charge-transfer complex, FTIR measurements were performed. The C≡N stretching bands observed at 2227 and 2214 cm^−1^ for neutral F4TCNQ shifted to 2194 and 2157 cm^−1^, respectively, upon complex formation. Such pronounced downshifts indicate an increase in electron density on the F4TCNQ moiety resulting from electron transfer from MeS-TTF-Ts to F4TCNQ, supporting the formation of charge-transfer interactions.

Since the samples obtained in this study are supramolecular nanofibers rather than single crystals, the vibrational frequencies may be influenced by molecular packing and local molecular environments. Nevertheless, the characteristic absorption bands assigned to F4TCNQ^−^ and TTF^+^ in the UV–vis spectra, together with the pronounced downshifts of the C≡N stretching bands observed in the FTIR spectra, provide strong evidence for substantial electron transfer from MeS-TTF-Ts to F4TCNQ and suggest the formation of a highly charge-transferred state in the nanofibers.

### Electrical conductivity of nanofibers formed from the (MeS-TTF-Ts)(F4TCNQ)_3_ charge-transfer complex

**Figure 6:**
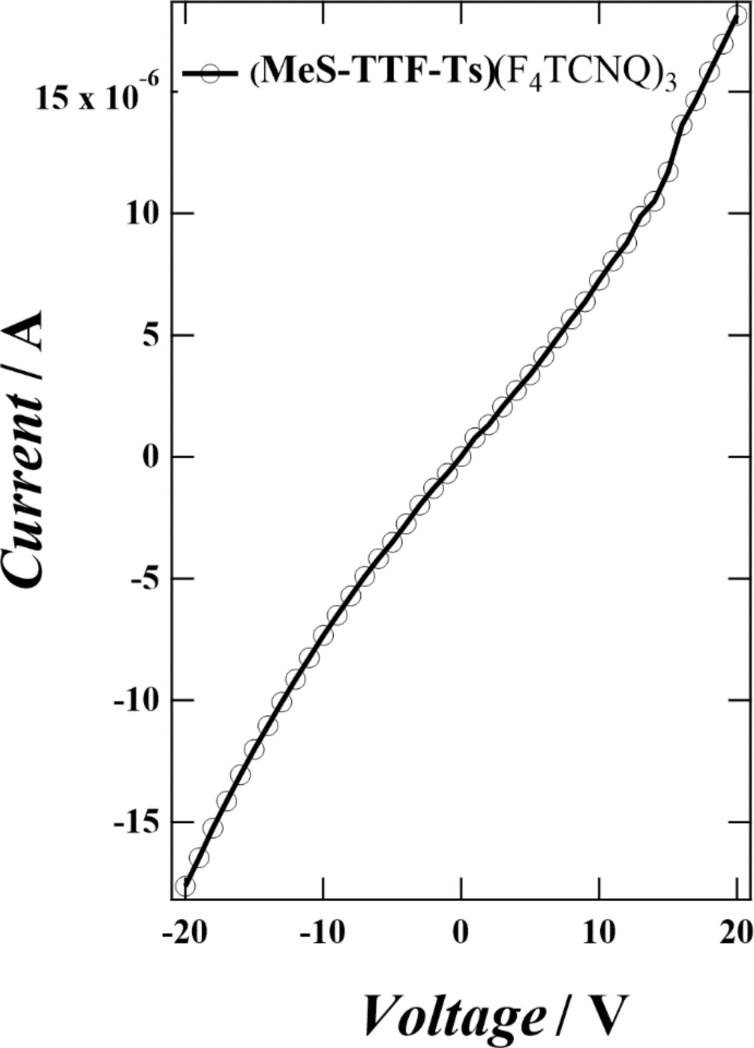
*I*–*V* curves of nanofibers composed of the (MeS-TTF-Ts)(F4TCNQ)_3_ charge-transfer complex deposited on a mica substrate.

Figure 6 shows the current–voltage (*I*–*V*) characteristics of nanofibers composed of the (MeS-TTF-Ts)(F4TCNQ)_3_ charge-transfer complex deposited on a mica substrate. The sample solution was prepared by mixing DMSO solution of MeS-TTF-Ts with an acetonitrile solution of F4TCNQ at a molar ratio of 1:3, with a final concentration of 6 mM.

The solution (4 μL) was cast onto a mica substrate equipped with gold electrodes (gap: 50 μm, electrode length: 6 mm) fabricated by vacuum deposition, followed by solvent evaporation to form nanofiber structures. Electrical conductivity measurements of the resulting nanofibers exhibited a clear current response, indicating good electrical conduction behavior. The electrical conductivity (σ) of the nanofibers composed of the (MeS-TTF-Ts)(F4TCNQ)_3_ complex was determined to be 1.24 × 10^−2^ S·cm^−1^. This result suggests that the one-dimensional stacked structure formed through donor–acceptor charge-transfer interactions functions as an effective charge transport pathway.

## Conclusion

In this study, a *C*_3_-symmetric discotic tetrathiafulvalene (TTF) derivative, MeS-TTF-Ts, was designed and synthesized as a molecular platform for constructing one-dimensional conductive nanostructures based on self-assembly. The molecular architecture features three TTF units radially arranged around a rigid *C*_3_-symmetric core and incorporates hydrogen-bonding sites, enabling the formation of highly ordered one-dimensional stacked structures through cooperative π–π stacking and directional intermolecular interactions.

Solution casting of MeS-TTF-Ts resulted in the formation of nanorod and nanofiber structures. These morphologies strongly depended on the solvent conditions, which influenced molecular organization and intermolecular packing during the self-assembly process. In particular, the use of DMSO resulted in the formation of uniform interconnected fibrous networks. These findings demonstrate that hierarchical control of supramolecular structures can be achieved through solvent modulation.

Furthermore, combining MeS-TTF-Ts with the strong electron acceptor F4TCNQ led to the formation of a charge-transfer complex, (MeS-TTF-Ts)(F4TCNQ)_3_, which successfully generated well-defined one-dimensional nanofiber structures. Spectroscopic measurements confirmed the formation of charge-transfer states, while morphological observations suggested the formation of one-dimensional stacked structures driven by donor–acceptor interactions. These results indicate that charge-transfer interactions can be effectively incorporated into self-assembled architectures through rational molecular design.

Electrical conductivity measurements revealed that the nanofibers exhibited a conductivity of 1.24 × 10^−2^ S·cm^−1^, demonstrating their potential as self-assembled organic nanowire materials. This conductivity is attributed to the one-dimensional stacked structure formed through donor–acceptor charge-transfer interactions, which functions as an efficient charge transport pathway.

Overall, this study demonstrates that the combination of molecular design and controlled self-assembly enables the construction of conductive supramolecular nanostructures with integrated structural and functional properties. These findings provide important insights into the relationship between molecular structure, supramolecular organization, and charge transport, and offer a useful design strategy for the development of organic conductive nanomaterials based on self-assembly.

## Supporting Information

File 1 Additional figures.

## Data Availability

All data that supports the findings of this study is available in the published article and/or the supporting information of this article.
