# Supplementary material for: Supramolecular one-dimensional conducting nanofibers from a C3-symmetric tetrathiafulvalene derivative
Source: Beilstein J Nanotechnol. 2026 Jul 10;17:872–81. doi: 10.3762/bjnano.17.63 (PMC13358902; doi:10.3762/bjnano.17.63)
Supplement: File 1 — Additional figures. [file Beilstein_J_Nanotechnol-17-872-s001.pdf]

BEILSTEIN JOURNAL OF NANOTECHNOLOGY

Supporting Information

for

Supramolecular one-dimensional conducting nanofibers from a C_3-symmetric tetrathiafulvalene derivative

Yoko Tatewaki, Fumiya Hirose, Sadafumi Nishihara, Tomoyuki Akutagawa, Takayoshi Nakamura and Tsuyoshi Minami

Beilstein J. Nanotechnol. **2026,** *17,* 872–881. doi:10.3762/bjnano.17.63

Additional figures

License and Terms: This is a supporting information file under the terms of the Creative Commons Attribution License (https://creativecommons.org/licenses/by/4.0). Please note that the reuse, redistribution and reproduction in particular requires that the author(s) and source are credited and that individual graphics may be subject to special legal provisions.
The license is subject to the *Beilstein Journal of Nanotechnology* terms and conditions: (https://www.beilstein-journals.org/bjnano/terms)

Experimental Section

FTIR measurements.

FTIR spectra of MeS-TTF-Ts were recorded using a JASCO FT/IR-4200 spectrometer equipped with an ATR accessory. The measurements were performed on the powdered sample at room temperature.

SEM measurements.

For SEM observations, MeS-TTF-Ts was dissolved in DMF or DMSO at a concentration of 6 mM. The charge-transfer complex sample was prepared by mixing a DMF solution of MeS-TTF-Ts with an acetonitrile solution of F4TCNQ at a molar ratio of 1:3, and the final concentration was adjusted to 6 mM. The resulting solutions were drop-cast onto HOPG substrates and allowed to dry under ambient conditions to form cast films.

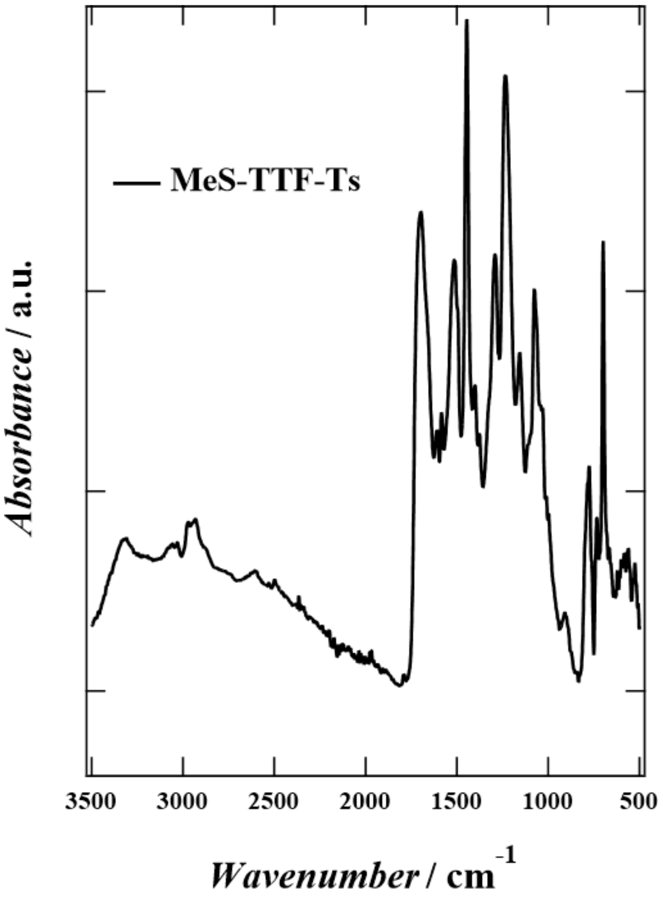

Figure S1: FTIR (ATR) spectrum of **MeS-TTF-Ts**.

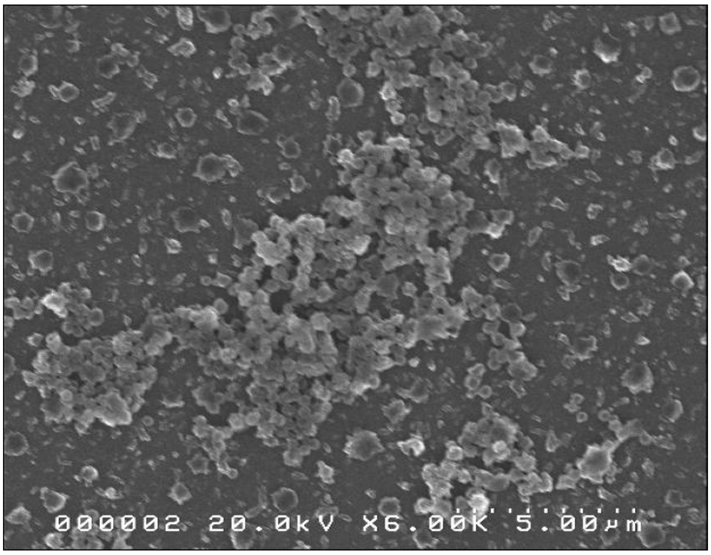

Figure S2: SEM image of a cast film of MeS-TTF-Ts deposited from a DMF solution on an HOPG substrate.

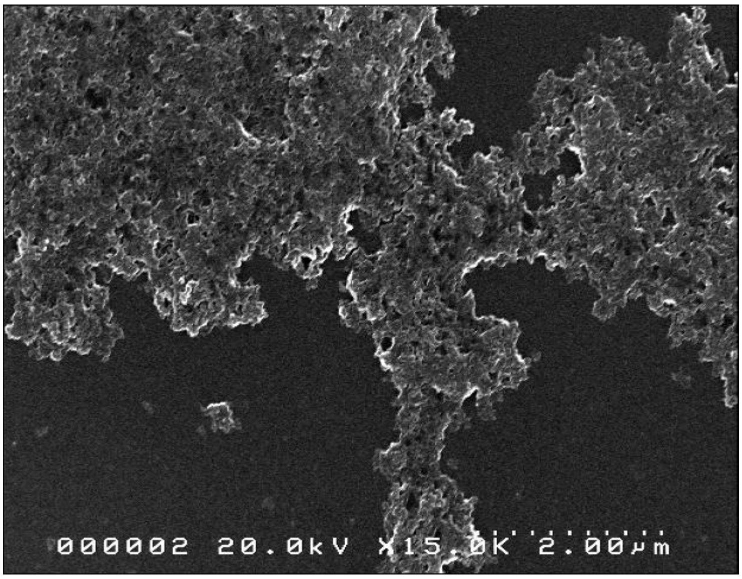

Figure S3: SEM image of a cast film of the C3-symmetric (**MeS-TTF-Ts**)($F4TCNQ)_3$ charge-transfer complex deposited from a mixed solution of DMF and acetonitrile on an HOPG substrate.

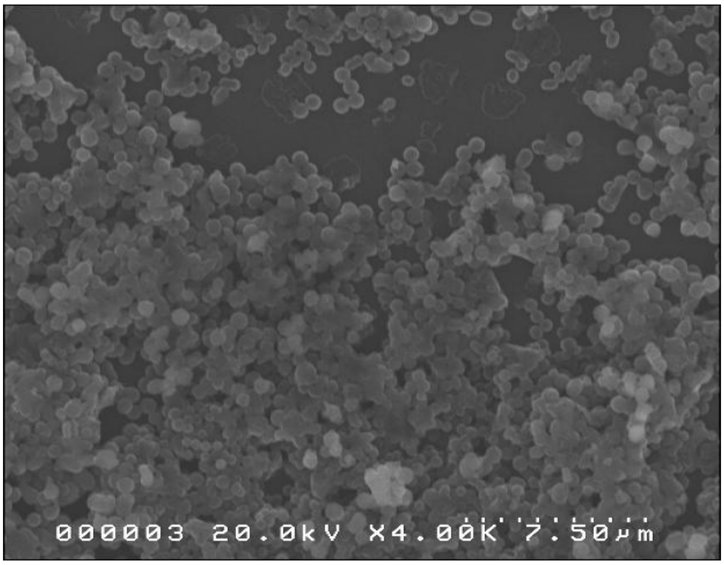

Figure S4: SEM image of a cast film of MeS-TTF-Ts deposited from a DMSO solution on an HOPG substrate.
